# Supplementary material for: Dynamical Majorana edge modes in a broad class of topological mechanical systems
Source: Nat Commun. 2017 Feb 23;8:14587. doi: 10.1038/ncomms14587 (PMC5331332; doi:10.1038/ncomms14587)
Supplement: Supplementary Information — Supplementary Figures 1 and Supplementary Note 1 [file ncomms14587-s1.pdf]

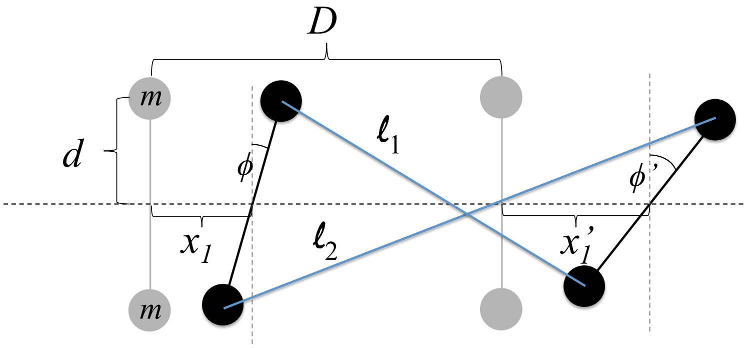

Supplementary Figure 1: The experimental setup. The shaded dimers represent the equilibrium configurations while the dark dimers represent a dynamical configuration. The blue oblique lines represent the springs. The figure highlights the degrees of freedom of each dimer. The parameters in the actual experiment are as follows: $M = 0.039$ kg, $d = 0.0278$ m and $D = 0.0484$ m. The constants of the springs were $K_1 = 200$ N/m and $K_2 \approx 400$ N/m. With these values, theory agrees reasonably well with the experiment, but not perfectly.

Supplementary Note 1: Analysis of Small Oscillations. In the following lines, we derive the dynamical matrix for the model described in Fig. 1 of the main document. This model is a theoretical representation of the mechanical system realized in laboratory and described in Fig. 2 of the main document. The purpose of the calculation is to demonstrate explicitly that, indeed, the phonon spectrum has the particle-hole symmetry. We will refer to the Supplementary Figure 1. Recall that, at equilibrium, all the springs are relaxed and the dimers assume a vertical position. The lengths of the two springs are denoted by $\ell_{1,2}$. When the springs are relaxed, they have the same length $\ell_0 = \sqrt{D^2 + 4d^2}$. The dimers have only two degrees of freedom, the displacement x_1 of the center of mass along the axis of the chain and the rotation of dimer, encoded in the degree of freedom $x_2 = d\phi$, where ϕ is the angle between dimer's axis and the vertical axis, and d is half the distance between the centers of the two masses of a dimer. Since the moment of inertia of a dimer is $I = 2md^2$, d equals the radius of gyration $d = \sqrt{I/M}$, $M = 2m$ being the total mass of a dimer.

Since we have only nearest-neighbors interactions, $q = 1$ and the potential appearing Eq. 1 of the main document is:

$$V_1(\mathbf{x}, \mathbf{x}') = \tfrac{1}{2}K_1(\ell_1 - \ell_0)^2 + \tfrac{1}{2}K_2(\ell_2 - \ell_0)^2 \ . \tag{1}$$

Simple geometrical considerations give:

$$\ell_1^2 = \left(D - x_1 - d\sin\tfrac{x_2}{d} + x_1' - d\sin\tfrac{x_2'}{d}\right)^2 + \left(d\cos\tfrac{x_2}{d} + d\cos\tfrac{x_2'}{d}\right)^2 , \tag{2}$$

$$\ell_2^2 = \left(D - x_1 + d\sin\tfrac{x_2}{d} + x_1' + d\sin\tfrac{x_2'}{d}\right)^2 + \left(d\cos\tfrac{x_2}{d} + d\cos\tfrac{x_2'}{d}\right)^2 . \tag{3}$$

To compute the $\widehat{Q}$ matrices, we need the second order derivatives of the potential, evaluated at the equilibrium configuration. Let ξ and ξ' be any of x_1, x_2, x_1' or x_2'. We have:

$$\frac{\partial V_1}{\partial \xi} = K_1 \frac{1}{2\ell_1}\frac{\partial \ell_1^2}{\partial \xi}(\ell_1 - \ell_0) + K_2 \frac{1}{2\ell_2}\frac{\partial \ell_2^2}{\partial \xi}(\ell_2 - \ell_0) \ . \tag{4}$$

Since the springs are uncompressed at the equilibrium, the second derivative must act on $\ell_{1,2} - \ell_0$, hence at equilibrium:

$$\frac{\partial^2 V_1}{\partial \xi \partial \xi'} = \frac{K_1}{4\ell_0^2}\frac{\partial \ell_1^2}{\partial \xi}\frac{\partial \ell_1^2}{\partial \xi'} + \frac{K_2}{4\ell_0^2}\frac{\partial \ell_2^2}{\partial \xi}\frac{\partial \ell_2^2}{\partial \xi'} \ . \tag{5}$$

The input for this equation is given below:

$$\frac{\partial \ell_1^2}{\partial x_1} = -2D, \quad \frac{\partial \ell_1^2}{\partial x_2} = -2D, \quad \frac{\partial \ell_1^2}{\partial x_1'} = +2D, \quad \frac{\partial \ell_1^2}{\partial x_2'} = -2D, \tag{6}$$

$$\frac{\partial \ell_2^2}{\partial x_1} = -2D, \quad \frac{\partial \ell_2^2}{\partial x_2} = +2D, \quad \frac{\partial \ell_2^2}{\partial x_1'} = +2D, \quad \frac{\partial \ell_2^2}{\partial x_2'} = +2D. \tag{7}$$

We can now assemble the $\widehat{Q}$ matrices $[\eta = (K_1 - K_2)/(K_1 + K_2)]$:

$$\widehat{Q}_0 = (K_1 + K_2)\frac{2D^2}{\ell_0^2}\begin{pmatrix} 1 & 0 \\ 0 & 1 \end{pmatrix}, \quad \widehat{Q}_{\pm 1} = (K_1 + K_2)\frac{2D^2}{\ell_0^2}\begin{pmatrix} -1 & \pm\eta \\ \mp\eta & 1 \end{pmatrix}. \tag{8}$$

Furthermore, the kinetic energy of one dimer has translational and rotational components and takes the form:

$$T = m\dot{x}_1^2 + m\dot{x}_2^2 \, . \tag{9}$$

Lastly, the dispersion equation takes the form:

$$(\omega^2 - \omega_0^2)\boldsymbol{A} = \omega_0^2 \begin{pmatrix} -\cos k & i\eta \sin k \\ -i\eta \sin k & \cos k \end{pmatrix} \boldsymbol{A} \, , \quad \omega_0 = \frac{D}{\ell_0}\sqrt{\frac{K_1 + K_2}{m}} \, . \tag{10}$$

We now can see explicitly that $k \to -k$ under the conjugation of the righthand side by $\widehat{\sigma_3}$, which is the manifestation of the inversion symmetry discussed in the main document. Furthermore, $\omega^2 - \omega_0^2 \to -(\omega^2 - \omega_0^2)$ under the conjugation by $\widehat{\sigma_1}$, that is, the particle-hole symmetry relative to ω_0^2 is indeed present as predicted by the general theory. The phonon spectra reported in Fig. 1 of the main document were generated with Supplementary Eq. (10). The data in panel (b) was generated with the experimental values of the parameters and is in good agreement with the experimental measurements reported in Fig. 2.
